# IFIT5 Participates in the Antiviral Mechanisms of Rainbow Trout Red Blood Cells

**DOI:** 10.3389/fimmu.2019.00613

**Published:** 2019-04-16

**Authors:** Veronica Chico, Maria Elizabhet Salvador-Mira, Ivan Nombela, Sara Puente-Marin, Sergio Ciordia, María Carmen Mena, Luis Perez, Julio Coll, Fanny Guzman, Jose Antonio Encinar, Luis Mercado, Maria del Mar Ortega-Villaizan

**Affiliations:** ^1^Departamento de Bioquímica y Biología Molecular, Instituto de Biología Molecular y Celular (IBMC), Universidad Miguel Hernández (UMH), Elche, Spain; ^2^Departamento de Bioquímica y Biología Molecular, Instituto de Investigación, Desarrollo e Innovación en Biotecnologîa Sanitaria de Elche (IDiBE), Universidad Miguel Hernández (UMH), Elche, Spain; ^3^Unidad de Proteómica, Centro Nacional de Biotecnología (CNB-CSIC), Madrid, Spain; ^4^Departamento de Biotecnología, Instituto Nacional de Investigaciones y Tecnologías Agrarias y Alimentarias (INIA), Madrid, Spain; ^5^Grupo de Marcadores Inmunológicos, Laboratorio de Genética e Inmunología Molecular, Instituto de Biología, Pontificia Universidad Católica de Valparaíso (PUCV), Valparaíso, Chile

**Keywords:** rainbow trout, IFIT5, red blood cells, erythrocyte, VHSV, antiviral immune response, immunoprecipitate, proteomic

## Abstract

Viral hemorrhagic septicemia virus (VHSV) infection appears to be halted in rainbow trout nucleated red blood cells (RBCs). Diverse mechanisms are thought to be related to the antiviral immune response of rainbow trout RBCs to VHSV. However, the specific rainbow trout RBC proteins that interact directly with VHSV are still unknown. In an attempt to identify VHSV-RBC protein interactions, we characterized the immunoprecipitated (IP) proteome of RBCs exposed to VHSV using an antibody against the N protein of VHSV. The IP proteomic characterization identified 31 proteins by mass spectrometry analysis. Among them, we identified interferon-induced protein with tetratricopeptide repeats 5 (IFIT5), a protein belonging to a family of proteins that are induced after the production of type I interferon. Importantly, IFIT5 has been implicated in the antiviral immune response. We confirmed the participation of IFIT5 in the rainbow trout RBC antiviral response by examining the expression profile of IFIT5 in RBCs after VHSV exposure at transcriptional and protein levels. We detected a correlation between the highest IFIT5 expression levels and the decline in VHSV replication at 6 h post-exposure. In addition, silencing *ifit5* resulted in a significant increase in VHSV replication in RBCs. Moreover, an increase in VHSV replication was observed in RBCs when the IFIT5 RNA-binding pocket cavity was modulated by using a natural compound from the SuperNatural II database. We performed a proximity ligation assay and detected a significant increase in positive cells among VHSV-exposed RBCs compared to unexposed RBCs, indicating protein-protein colocalization between IFIT5 and the glycoprotein G of VHSV. In summary, these results suggest a possible role of IFIT5 in the antiviral response of RBCs against VHSV.

## Introduction

The role of nucleated red blood cells (RBCs) as immune response cell mediators has become clearer in recent years. Fish RBCs are the most common cell type in the blood and are best known for their functions in gas exchange and respiration. In mammals, mature RBCs are oval, biconcave cells that lack nuclei, organelles, and ribosomes (1). In non-mammalian vertebrates, RBCs are oval, flattened, biconvex disks with a nucleus and organelles in their cytoplasm (2), allowing them to *de novo* synthesize molecules in response to stimuli. Indeed, a set of biological processes related to antiviral immunity has been described in nucleated RBCs (3).

Recent studies have demonstrated that RBCs halt viral hemorrhagic septicemia rhabdovirus (VHSV) infection (4). The virus enters the cell, but does not replicate at levels comparable to piscine orthoreovirus or infectious salmon anemia virus (ISAV) infections in salmon RBCs (5, 6). Several mechanisms have been suggested to be involved in the antiviral response of rainbow trout RBCs to VHSV, such as increased protein levels of β-defensin 1 (BD1, an antimicrobial peptide involved in antiviral innate immunity), global protein synthesis inhibition corresponding to a virus and host cell shut-off, or an antioxidant-related antiviral response (4). Further, an increase in the expression of IFN-related genes and proteins, such as Mx, has been observed in infectious pancreatic necrosis virus-exposed RBCs, where the viral infection was also non-productive (7).

Interferon-induced proteins with tetratricopeptide repeats (IFIT) are a family of proteins that are strongly induced after the production of type I interferon (8). The IFIT family is conserved in mammals, amphibians, birds, and bony fishes, but is not present in yeast, plants, or invertebrates (8). IFIT genes have been identified for many mammal species, as well as for various birds, reptiles, amphibians, and bony fishes. The number of IFIT genes and the composition of the family varies greatly between species (9). Knowledge of IFIT genes is scarce in teleost species compared to humans. Recently, the complete repertoire of IFIT genes was described in zebrafish (10). IFIT proteins contain repeats of tetratricopeptide (TPR domains) and mediate a wide variety of protein-protein and RNA-protein interactions involved in the initiation of translation, virus replication, double-stranded RNA signaling, cell migration, and proliferation (11, 12). The multiple TPR domains may allow IFIT proteins to participate in the regulation of viral gene transcription and translation and in the negative regulation of the host inflammatory and antiviral response (12, 13). Recently, it has been shown that members of the IFIT family selectively restrict viral replication by recognition of viral mRNA via binding of 2'-O methylated RNA or 5'-triphosphate RNA (14–16). Other studies have found that members of the IFIT family decreased host cap-dependent protein translation by binding to the subunits of the eukaryotic initiation factor 3 (eIF3) translation complex (17). Studies conducted using double hybrid technique (Y2H) also suggested that IFIT proteins could bind to other viral proteins. For example, IFIT1 has been described to bind E1, a viral helicase of human papilloma virus (HPV) required for replication, sequestering it within the cytoplasm and preventing viral replication in the nucleus (18).

In an attempt to elucidate the molecules responsible for halting VHSV replication inside rainbow trout RBCs, we characterized the RBC proteome that immunoprecipitates with the N protein of VHSV after RBC exposure to the virus. The IFIT5 protein was identified, and additional experiments confirmed the participation of IFIT5 in the rainbow trout RBC antiviral response. We determined the expression profile of IFIT5 in VHSV-exposed RBCs and detected a correlation between the highest IFIT5 expression level and diminished VHSV replication at 6 h post exposure (hpe) to VHSV. In addition, silencing *ifit5* resulted in a significant increase in VHSV replication in RBCs. IFIT5 modulation activity assays were performed by modulating the IFIT5 RNA-binding pocket cavity using a natural compound from the SuperNatural II (19) database identified through a molecular docking procedure. VHSV replication increased in VHSV-exposed RBCs when the IFIT5 RNA-binding pocket cavity was modulated by the compound. Moreover, a proximity ligation assay (PLA) was performed to investigate possible protein-protein colocalization between IFIT5 and the glycoprotein G of VHSV (GVHSV). A significant increase in positive cells was detected among VHSV-exposed RBCs compared with unexposed RBCs. Altogether, the results support a potential role of IFIT5 in the antiviral response of RBCs against VHSV.

## Materials and Methods

### Animals

Rainbow trout (*Oncorhynchus mykiss*) >6 to 7 cm were obtained from Piszolla S.L., Cimballa Fish Farm, (Zaragoza, Spain) and maintained at the University Miguel Hernandez (UMH) facilities at 14°C. Before experimentation, fish were acclimatized to laboratory conditions for 2 weeks. All experimental protocols were reviewed and approved by the Animal Welfare Body and the Research Ethics Committee at the UMH and by the competent authority of the Regional Ministry of Presidency and Agriculture, Fisheries, Food and Water supply. Animal care and all activities involving animal handling and experiments were done according to Spanish [Real Decreto 1201/2005] and EU [EU Directive EC86/609, and Appendix A to Convention ETS123, 2007/526/CE] regulations and recommendations for animal experimentation.

### Cell Cultures and Virus

Rainbow trout RBCs were obtained and purified as previously described (4, 7). Briefly, RBCs extracted from the caudal vein were purified by 2 successive Ficoll density gradient centrifugations (7,206 g, Ficoll 1.007; Sigma-Aldrich, Madrid, Spain). Ficoll-purified RBCs were maintained in 25 cm^2^ flasks (Nunc Roskilde, Denmark) with RPMI-1640 medium (Dutch modification) (Gibco, Thermo Fisher Scientific, Carlsbad, CA) supplemented with 10% fetal bovine serum (FBS) gamma irradiated (Cultek, Madrid, Spain), 1 mM pyruvate (Gibco), 2 mM L-glutamine (Gibco), 50 μg/mL gentamicin (Gibco), 2 μg/mL fungizone (Gibco), 100 U/mL penicillin (Sigma-Aldrich), and 100 μg/mL streptomycin (Sigma-Aldrich) at 14°C for 24 h prior to experimentation.

The RTG-2 (rainbow trout gonad-2) cell line was purchased from the American Type Culture Collection (ATCC 50643) and maintained at 21°C in MEM medium (Sigma-Aldrich) containing 10% FBS, 1 mM pyruvate, 2 mM L-glutamine, 50 μg/mL gentamicin, and 2 μg/mL fungizone.

VHSV strain 07.71 (20) was purchased from the American Type Culture Collection (ATCC VR-1388) and cultured in fathead minnow epithelioma papulosum cyprini (EPC) (21) cells at 14°C as previously described (22).

### Immunoprecipitation Assay

Ficoll-purified RBCs from 3 fish were exposed to VHSV at multiplicity of infection (MOI) 1 for 3 h as previously described (4). Unexposed RBCs were used as a control. RBCs were washed 3 times with phosphate-buffered saline (PBS), and the pellet was resuspended in RIPA buffer (50 mM Tris-HCl [Sigma-Aldrich] pH 7.4, 150 mM NaCl [Sigma-Aldrich], 1% Triton X-100 [Sigma-Aldrich], 1% sodium deoxycholate [Sigma-Aldrich], 0.1% sodium dodecyl sulfate [SDS; NZY Tech, Genes and Enzymes, Lisbon, Portugal], 1 mM EDTA [Sigma-Aldrich]) with a cocktail of protease inhibitors (Sigma-Aldrich). The cell pellet was homogenized using pestles (Invitrogen, Thermo Fisher Scientific) and kept at−20°C until use. Cell lysate was precleared with 50 μL protein A Sepharose 4 Fast Flow (GE Healthcare, Little Chalfont, UK) for 1 h at 4°C. Samples were centrifuged at 12,000 x g for 20 s, and supernatants were incubated with monoclonal murine 2C9 antibody against N protein of VHSV (23) diluted 1/500 overnight at 4°C. Immune complexes were precipitated by adding 50 μL protein A Sepharose 4 Fast Flow (GE Healthcare) for 1 h at 4°C. After washing the pellet with RIPA buffer, immune complexes were dissociated by heating the samples in 2X loading sample buffer (from 5X loading sample buffer: 0.225 M Tris-Cl [pH 6.8], 50% glycerol [Sigma-Aldrich], 5% SDS, 0.05% bromophenol blue [Panreac, Barcelona, Spain], and 0.25 M dithiothreitol (Invitrogen, Thermo Fisher Scientific) for 3 min at 95°C. After centrifugation at 12,000 x g for 20 s, we analyzed the supernatant by mass spectrometry.

### In-gel Protein Digestion

Each immunoprecipitated sample was eluted with 2X loading sample buffer and then applied into 1.2 cm wide wells of a conventional SDS-PAGE gel (1 mm thick, 4% acrylamide for the stacking gel, and 12% acrylamide for the resolving gel). The run was stopped as soon as the front was 1 cm into the resolving gel, so that the whole proteome was concentrated in the stacking/resolving gel interface. The unseparated protein bands were visualized by Coomassie staining, excised, cut into 1 mm^2^ cubes, deposited in 96 well plates, and processed automatically in a Proteineer DP (Bruker Daltonics, Bremen, Germany). The digestion protocol was based on Schevchenko et al. (24) with minor variations: gel plugs were washed first with 50 mM ammonium bicarbonate and second with ACN prior to reduction with 10 mM DTT in 25 mM ammonium bicarbonate solution, and alkylation was carried out with 55 mM IAA in 50 mM ammonium bicarbonate solution. Gel pieces were rinsed first with 50 mM ammonium bicarbonate and second with ACN and then were dried under a stream of nitrogen. Proteomics-grade Trypsin (Sigma-Aldrich) at a final concentration of 16 ng/μL in 25% ACN/50 mM ammonium bicarbonate solution was added, and the digestion took place at 37°C for 4 h. The reaction was stopped by adding 50% ACN/0.5% TFA for peptide extraction. The tryptic eluted peptides were dried by speed-vacuum centrifugation and were cleaned/desalted using Stage-Tips with Empore 3M C18 disks (Sigma-Aldrich).

### Liquid Chromatography and Mass Spectrometry Analysis

A 1 μg aliquot of each digested sample was subjected to 1D-nano LC ESI-MSMS analysis using a nano liquid chromatography system (Eksigent Technologies nanoLC Ultra 1D plus, SCIEX, Foster City, CA) coupled to a high-speed Triple TOF 5600 mass spectrometer (SCIEX) with a Nanospray III source. The analytical column was a silica-based, reversed-phase Acquity UPLC® M-Class Peptide BEH C18 Column, 75 μm × 150 mm, 1.7 μm particle size, and 130 Å pore size (Waters Corporation, Milford, MA, USA). The trap column was a C18 Acclaim PepMapTM 100 (Thermo Fisher Scientific), 100 μm × 2 cm, 5 μm particle diameter, 100 Å pore size, switched online with the analytical column. The loading pump delivered a solution of 0.1% formic acid in water at 2 μL/minutes. The nano-pump provided a flow rate of 250 nL/minutes and was operated under gradient elution conditions. Peptides were separated using a 100-min gradient ranging from 2 to 90% mobile phase B (mobile phase A: 2% acetonitrile, 0.1% formic acid; mobile phase B: 100% acetonitrile, 0.1% formic acid). Injection volume was 5 μL.

Data acquisition was performed with a TripleTOF 5600 System (SCIEX). Data were acquired using an ionspray voltage floating (ISVF) 2300 V, curtain gas (CUR) 35, interface heater temperature (IHT) 150, ion source gas 1 (GS1) 25, and declustering potential (DP) 100 V. All data were acquired using information-dependent acquisition (IDA) mode with Analyst TF 1.7 software (SCIEX). For IDA parameters, 0.25 s MS survey scans in the mass range of 350–1,250 Da were followed by 35 MS/MS scans of 100 ms in the mass range of 100–1,800 (total cycle time: 4 s). Switching criteria were set to ions greater than mass-to-charge ratio (m/z) 350 and smaller than m/z 1,250 with charge state of 2-5 and an abundance threshold of >90 counts per second (cps). Former target ions were excluded for 15 s. An IDA rolling collision energy (CE) parameters script was used to automatically control the CE.

### Proteomic Data Analysis, Sequence Search, and Protein Network Analysis

Mass spectrometry data obtained were processed using PeakView v2.2 Software (SCIEX) and exported as mgf files that were searched using Mascot Server v2.5.1 (Matrix Science, London, UK) against a protein database including Teleostei protein sequences from Uniprot/Swissprot Knowledgebase (last update: 20170412, 2,542,118 sequences), together with commonly occurring contaminants, as previously described (25).

Search parameters were set as follows: enzyme, trypsin; allowed missed cleavages, 2; and carbamidomethyl (C) as fixed modification and acetyl (Protein N-term), pyrolidone from E, pyrolidone from Q and oxidation (M) as variable modifications. Peptide mass tolerance was set to ±25 ppm for precursors and 0.05 Da for fragment masses. The confidence interval for protein identification was set to ≥95% (*P* < 0.05), and only peptides with an individual ion score above the 1% false discovery rate (FDR) at spectra level were considered correctly identified.

Protein-protein interaction (PPI) networks were analyzed using STRING v10.5 (http://string.embl.de/) (26), with a medium confidence threshold score of 0.4. The *Homo sapiens* model organism was used for the analysis. Gene symbols were obtained through sequence homology with *H. sapiens* orthologs using Blast2GO version 4.1.9.

### Shotgun Proteomics for IFIT5 Peptide Validation

Nano LC ESI-MSMS analysis was performed using an Eksigent 1D-nanoHPLC coupled to a 5600 TripleTOF QTOF mass spectrometer (SCIEX). The analytical column was a silica-based, reversed-phase nanoACQUITY UPLC 75 μm × 15 cm, 1.7 μm particle size (Waters Corporation, Milford, MA, USA). The trap column was an Acclaim PepMap 100, 5 μm particle diameter, 100 Å pore size, switched online with the analytical column. The loading pump delivered a solution of 0.1% formic acid in 98% water/2% acetonitrile (Scharlab, Barcelona, Spain) at 3 μL/min. The nanopump provided a flow rate of 250 nL/min and was operated under gradient elution conditions using 0.1% formic acid (Fluka, Buchs, Switzerland) in water as mobile phase A and 0.1% formic acid in 100% acetonitrile as mobile phase B. Gradient elution was performed according to the following scheme: isocratic conditions of 96% A:4% B for 5 min, linear increase to 40% B at 25 min, linear increase to 95% B after 2 min, isocratic conditions of 95% B for 5 min, and return to initial conditions after 10 min. Injection volume was 5 μL. The LC system was coupled via a nanospray source to the mass spectrometer. Automatic data-dependent acquisition using dynamic exclusion allowed both full scans of (m/z 350-1,250) MS spectra and tandem MS CID spectra of the 15 most abundant ions. Acquisition time was 250 and 100 ms for MS and MSMS spectra, respectively. The candidate peptide was synthesized using standard Fmoc chemistry in an Intavis Multiple peptide synthesizer (INTAVIS, Cologne, Germany). The synthetic peptide was used to confirm the peptide sequence identified by shotgun proteomics.

### Time Course of *ifit5* Expression in Rainbow Trout RBCs and RTG-2 Exposed to VHSV

Ficoll-purified RBCs (10^6^ cells/well) and RTG-2 cells (2.5^*^10^5^ cells/well) were exposed to VHSV at MOI 1 at different time points (0, 3, 6, 24, and 72 h). Cells were resuspended and stored in appropriate buffer for RNA extraction. The expression levels of NVHSV and the *ifit5* gene were analyzed by real time RT-qPCR.

### Rainbow Trout Anti-IFIT5 Antibody Production

Rainbow trout anti-IFIT5 antibody was designed and produced at Dr. Luis Mercado's laboratory. First, antigenic propensity from different regions of the rainbow trout IFIT5 protein sequence (GenBank: AAM18469.1) were identified, analyzing hydrophobicity (Hopp & Woods test), accessibility, and flexibility. Then, peptides from the most antigenic regions were selected for peptide synthesis. Before synthesis, peptide sequences were modified by replacing cysteine residues with serine residues to avoid formation of intrachain disulfide bonds. Cysteine was added to the start and end of the peptide sequence to produce dimers. Peptide synthesis was performed using mesh packets with resin as previously described (27). Peptide cleavage was performed using cold petroleum ether. After cleavage of the peptide from the resin, peptides were characterized by MALDI-TOF (Bruker Daltonics Inc, Billerica, MA). Oxidation of peptides was performed by dilution of the peptide in miliQ water and agitating overnight. After oxidation, peptides were purified using G10 resin (Sigma-Aldrich) to eliminate salts.

To immunize mice against the peptides, 100 μg of oxidized peptide was conjugated with Freund's adjuvant (Thermo Fisher Scientific) following the manufacturer's instructions. The mix was subcutaneously injected into mice. A total of 3 injections were made with a lapse of 2 weeks between them. After all immunizations were done, euthanasia was carried out with anesthetic overdose. Blood was collected by cardiac injection. Blood sera were obtained by letting blood coagulate for 30 min at 37°C followed by an incubation at 4°C for 1 h. Finally, blood was centrifuged at 5,000 rpm for 10 min, and serum was collected. An ELISA was performed to determine the serum titers (Supplementary Figure 1). Wells were coated with 100 ng of the corresponding peptides diluted in 100 μL of PBS overnight at 4°C. Blank wells were coated using only PBS. Then, PBS with peptides was removed and wells were washed 3 times with PBS. Then, 5% non-fat milk was used to block the wells at 37°C in agitation for 2 h. Sera were diluted in PBS (from 1/500 to 1/64,000) and incubated at 37°C for 90 min. Wells were further washed 3 times and incubated with goat anti-mouse HRP-conjugated antibody diluted 1/7,000 for 1 h at 37°C in agitation. Each well was incubated with 100 μL of 3,3',5,5'-tetramethylbenzidine (Thermo Fisher Scientific) for 15 min. The reaction was stopped with 50 μL 1 N sulfuric acid (Winkler Ldta, Santiago, Chile). Plates were read at 450 nm using VersaMax™ Tunable microplate reader (Molecular Devices LLC, San Jose, CA, USA). Anti-IFIT5 antibody was validated by western blot (Supplementary Figure 1). RBC cell pellets were homogenized and applied into Tris-Glycine sodium dodecyl sulfate 12% polyacrylamide gels under reducing conditions as previously described (4). Briefly, proteins in the gel were transferred to a nitrocellulose membrane (BioRad, Madrid, Spain) and blocked with 8% dry milk and 1% Tween-20 in PBS. Then, the membrane was incubated with the antibody diluted 1/500 in PBS containing 0.5% dry milk and 0.5% Tween-20 (PMT buffer), overnight at 4°C. Finally, the membrane was washed and incubated with secondary antibody goat anti-mouse peroxidase (Sigma-Aldrich) in PMT buffer for 45 min, and proteins were detected with ECL chemiluminescence reagents (Amersham Biosciences, Buckinghamshire, UK) and revealed by exposure to an X-ray film.

### Flow Cytometry and Immunofluorescence of IFIT5

RBCs were fixed, permeabilized, and incubated with primary and secondary antibodies as previously described (4). Mouse anti-IFIT5 antibody at 1/300 dilution in permeabilization buffer and rabbit anti-GVHSV antibody (kindly provided by Dr. Neils Lorenzen to Dr. Julio Coll) at 1/300 dilution in permeabilization buffer were used as primary antibodies. Secondary antibodies used in these studies included anti-mouse IgG CF^TM^ 488 and anti-rabbit IgG CF^TM^ 647 (Sigma-Aldrich) produced in goat. Flow cytometry analysis was performed using a FACSCanto II (BD Biosciences, Madrid, Spain) flow cytometer. A total of 30,000 events were adquired. Gating selection for RBCs is shown in Supplementary Figure 2. To obtain immunofluorescence images, cells were incubated with 4′,6-diamidine-2′-phenylindole dihydrochloride (DAPI) (Sigma-Aldrich) at 0.3 pg/mL for 5 min to stain the cell nucleus. Immunofluorescence images were taken with the IN Cell Analyzer 6000 (GE Healthcare).

### Molecular Docking Procedure to Search for IFIT5 Modulators

The crystal structure of the IFIT5 protein has been solved in human (28), but not rainbow trout. Therefore, we constructed a model using rainbow trout IFIT5 amino acid sequences (GenBank AAM18469.1) and human IFIT5 protein as template. A homology modeling approach for rainbow trout IFIT5 was performed using the Swiss-Model server (29) and the crystal structure of human IFIT5 (PDB number 5UDL).

The crystal structure of IFIT5 showed an RNA-binding pocket cavity (28). Therefore, we searched for compounds capable of binding to this cavity to modulate its functions. The screen resulted in a list of 325,508 natural compounds from the SuperNatural II database (19). Mol2 files were converted into pdbqt format using the Python script “prepare_ligand4.py” included in the AutoDockTools-1.5.7.rc1 (30, 31).

Prior to the docking procedure, the protein (receptor) and ligand structures were prepared as previously described (30, 31). The IFIT5 modeled protein structure was subjected to geometry optimization using the repair function of the FoldX algorithm (32). The docking procedure was performed with AutoDock/Vina (33) as described (31, 34). Compounds with the lowest calculated Gibbs free energy variations (ΔG ≤-11 kcal/mol) were selected as putative modulators.

### *In silico* Analysis of Pharmacokinetic Parameters and Toxicity Potential Properties of the Modulator Candidates

DataWarrior v4.2.2 software (Allschwil, Switzerland) (35) was used to calculate molecular descriptors, such as the topological polar surface area (TPSA), molecular weight (MW), estimated logarithm (base 10) of the solubility measured in mol/L (logS), estimated logarithm (base 10) of the partition coefficient between n-octanol and water (logP), number of hydrogen bond acceptors, number of hydrogen bond donors, violations of Lipinski's rule of 5 (36), drug score, and fragment-based druglikeness. The *in silico* absorption, distribution, metabolism, excretion, and toxicity (ADMET) properties of all compounds were calculated using admetSAR (37) and DataWarrior v.4.2.2 software. The selected compounds were purchased from the chemical supplier MolPort (MolPort SIA, Riga, Latvia).

### Modulating IFIT5 Activity With Selected Compounds

The compounds were resuspended in dimethyl sulfoxide (DMSO) (Sigma-Aldrich) at a concentration of 10 mM and diluted in RPMI 10% FBS to concentrations ranging from 540 to 4,860 nM. To evaluate the toxicity of the compounds in RBCs, the LIVE/DEAD® Cell Vitality Assay Kit (Thermo Fisher Scientific) was used according to the manufacturer's instructions. Briefly, RBCs were incubated with different concentrations of the compounds in RPMI 10% FBS for 24 h at 14°C. Then, LIVE/DEAD imaging reagents were mixed at equal volumes and added to the cells and incubated for 15 min at room temperature. Samples were analyzed using the FACSCanto II (BD Biosciences) flow cytometer. Live cells fluoresce green (calcein AM substrate), and dead cells fluoresce red (ethidium homodimer-1 [EthD-1]). Each subpopulation was separated into 4 quadrants. The percentage of cell viability was calculated as follows: [number of green-stained cells in treated RBCs/number of green-stained cells in non-treated RBCs] x 100. A total of 20,000 events were adquired. RBCs treated with 100 μM of H_2_0_2_ (Sigma-Aldrich) were used as a positive control for dead RBCs.

To evaluate IFIT5 activity modulation by the compounds, RBCs were incubated with 4,860 nM of the compounds for 24 h. After removing the compounds, cells were washed with culture medium and then exposed to VHSV at MOI 1 for 24 h at 14°C. Samples were stored in buffer for RNA extraction, and RT-qPCR was performed.

### Modulating IFIT5 RNA-binding Pocket Cavity With VHSV RNA

RNA was isolated from VHSV (4^*^10^8^ PFU/mL) as described below. Then, RBCs were electroporated with 5 μL VHSV RNA using the Neon™ Transfection System (Life Technologies, Thermo Fisher Scientific), at 1,600 V, 30 ms, and 1 pulse. After 24 h of incubation, RBCs were washed with culture medium and exposed to VHSV MOI 1. After 3 h of incubation at 14°C, samples were washed with culture medium and some samples were stored in buffer for RNA extraction while others were further incubated for 24 h. The cell pellets of these samples were stored in RNA extraction buffer.

### IFIT5 Small Interfering RNA (siIFIT5) Assay

Three different IFIT5 small interfering RNA sequences (siIFIT5) were designed and produced by Sigma-Aldrich (Table 1). RBCs were transfected with a mixture of 3 siIFIT5 sequences by electroporation. For each electroporation reaction, we used 187 pmol of each siRNA per 0.5^*^10^6^ cells resuspended in Buffer T (Neon™ Transfection System Kit, Life Technologies). As a negative control, 187 pmol siGFP was used (Sigma-Aldrich). RBCs were electroporated as described above and incubated for 3 days at 14°C.

**Table 1:** Sequences of rainbow trout *ifit5*-specific siRNA.

**Name**	**siRNA design sequence (5′- 3′)**	**Start on target**
siIFIT5-1 sense	GGUAUUCCAAGGGCCUCAAdTdT	432
siIFIT5-1 antisense	UUGAGGCCCUUGGAAUACCdTdT	432
siIFIT5-2 sense	CAAUGAGUCCCUACACAUUdTdT	857
siIFIT5-2 antisense	AAUGUGUAGGGACUCAUUGdTdT	857
siIFIT5-3 sense	CAGCUUACCUUCAGUACAUdTdT	461
siIFIT5-3 antisense	AUGUACUGAAGGUAAGCUGdTdT	461

IFT5 silencing was evaluated at transcript and protein level. *ifit5* gene silencing was analyzed by RT-PCR, described in section RNA Isolation and Gene Expression by RT-PCR and RT-qPCR. Moreover, IFIT5 silencing was determined by western blot, described in section Rainbow Trout Anti-IFIT5 Antibody Production, using anti-IFIT5 antibody at 1/500 dilution and anti-α-actin antibody (Sigma-Aldrich) (1/100) was used as endogenous control. Goat anti-mouse peroxidase and goat anti-rabbit peroxidase (Sigma-Aldrich), were respectively used for anti-IFIT5 and anti-α-actin antibodies.

Transfected RBCs were separately exposed to VHSV MOI 1 at 14°C. After 3 h of VHSV exposure, RBCs were washed with culture medium and incubated for 24 h. Cells were resuspended and stored in appropriate buffer for RNA extraction.

### RNA Isolation and Gene Expression by RT-PCR and RT-qPCR

RNA was isolated using the E.Z.N.A.® Total RNA Kit (Omega Bio-Tek Inc., Norcross,GA). Subsequent DNAse treatment was done using TURBO™ DNase (Ambion, Thermo Fisher Scientific) as previously described (4). Then, cDNA synthesis from RNA samples were performed as described in Chico et al. (38) using M-MLV reverse transcriptase (Invitrogen, Thermo Fisher Scientific).

To evaluate *ifit5* gene silencing, cDNA from siRNA-treated samples were analyzed by semiquantitative RT-PCR. PCR amplification reactions were performed as follows: 0.5 μL dNTP mix (10 mM each) (Invitrogen, Thermo Fisher Scientific), 0.125 μL GoTaq® DNA polymerase (Promega Biotech, Madrid, Spain), 5 μL 5X Green GoTaq® reaction buffer (Promega Biotech), 0.5 μL each primer (20 μM) (Table 2), and 2.5 μL cDNA in a total volume of 25 μL. PCRs were carried out in a GeneAmp® PCR System 2700 thermocycler (Applied Biosystems, Thermo Fisher Scientific). Cycling conditions were 94°C for 5 min followed by 30 cycles at 94°C for 1 min, specific annealing temperature (Table 2) for 1 min and 72°C for 90 s. Then, 1 cycle at 72°C was held for 7 min. Glyceraldehyde 3-phosphate dehydrogenase (GAPDH) (39) (Table 2) was used as a housekeeping gene to normalize the RT-PCR. PCR products were visualized on a 1.2% agarose gel stained with Gelred® nucleic acid stain (Biotium, Inc. Fremont CA, USA). NVHSV and *ifit5* genes were analyzed by real-time RT-qPCR. RT-qPCR was carried out using the ABI PRISM 7300 System (Thermo Fisher Scientific). Cycling conditions and gene expression analysis methods have been previously described (4). Primers and probes are listed in Table 3. The *ef1*α and eukaryotic 18S rRNA genes (Cat#4310893E, Thermo Fisher Scientific) were used as endogenous controls.

**Table 2:** Sequences of primers and probes for RT-PCR.

**Gene**	**Forward primer (5′- 3′)**	**Reverse primer (5′- 3′)**	**Annealing T^a^ (C°)**	**Reference or** **accession number**
*ifit5*	TCTACAGGGGGAGCCAAACA	AGGGCTAGGAGGACCATGAC	60	AF483530.1
*gapdh*	ATGTCAGACCTCTGTGTTGG	TCCTCGATGCCGAAGTTGTCG	52	(39)

**Table 3:** Sequences of primers used for RT-qPCR.

**Gene**	**Forward primer (5′- 3′)**	**Reverse primer (5′- 3′)**	**Probe (5′-3′)**	**Reference or** **accession number**
*ifit5*	CCCTCAATGACTCTGACAAGCA	CCCTGCCCTCATCTTTCTTCT	CCAGCTTCGGCCTGTTTCTGTTCCA	(40)
NVHSV	GACTCAACGGGACAGGAATGA	GGGCAATGCCCAAGTTGTT	TGGGTTGTTCACCCAGGCCGC	(38)
*ef1α*	ACCCTCCTCTTGGTCGTTTC	TGATGACACCAACAGCAACA	GCTGTGCGTGACATGAGGCA	(41)

### *In situ* Proximity Ligation Assay (PLA)

Ficoll-purified RBCs from rainbow trout blood were exposed to VHSV MOI 1 for 6 h at 14°C. Unexposed and VHSV-exposed RBCs were attached to frosted slides (Thermo Fisher). The attached cells were fixed with 4% paraformaldehyde (PFA) for 1 h at room temperature. Then, samples were washed 3 times with PBS. The slides were incubated with 70% ethanol for 30 s, placed on ice for 60 min, and air-dried. Finally, slides were stored at −20°C until PLA. PLA recognizes close-proximity proteins (40 nm) by primary antibodies, which are then recognized by secondary antibodies conjugated to specific oligonucleotides. Subsequently, a ligase and complementary oligonucleotides are added and a circle is form, which is amplified by rolling circle amplification. The amplification product is detected with fluorescent oligonucleotides (42). The PLA was performed using Duolink® *in situ*-Fluorescence kit (Sigma-Aldrich) following the manufacturer's instructions. Briefly, blocking solution was added to each sample, and the slides were incubated in a humidity chamber for 1 h at 37°C. After that, the blocking solution was tapped off and slides were incubated with a primary antibody against IFIT5 protein produced in mice and against GVHSV protein produced in rabbit diluted at 1/300 in a volume of 100 μL overnight at 4°C in a humidity chamber. Detection was performed using PLA secondary probes and ligation according to the manufacturer's instructions. Finally, the slides were mounted with a cover slip using Duolink *in situ* Mounting Medium with DAPI and stored at −20°C until analysis. All imaging pictures were taken with the IN Cell Analyzer 6000 (GE Healthcare). Positive cells were counted using a designed routine in the IN Cell Analyzer workstation 3.7.2 software.

### Software and Statistics

Graphpad Prism 6 software (www.graphpad.com) was used for statistics calculations and graphic representation. Statistic tests and associated *P*-values are indicated in each assay. Flow cytometry data were processed and analyzed using Flowing Software 2.5.1 (www.flowingsoftware.com/).

## Results

### Immunoprecipitated Proteins From VHSV-exposed RBCs

**Figure 1:**
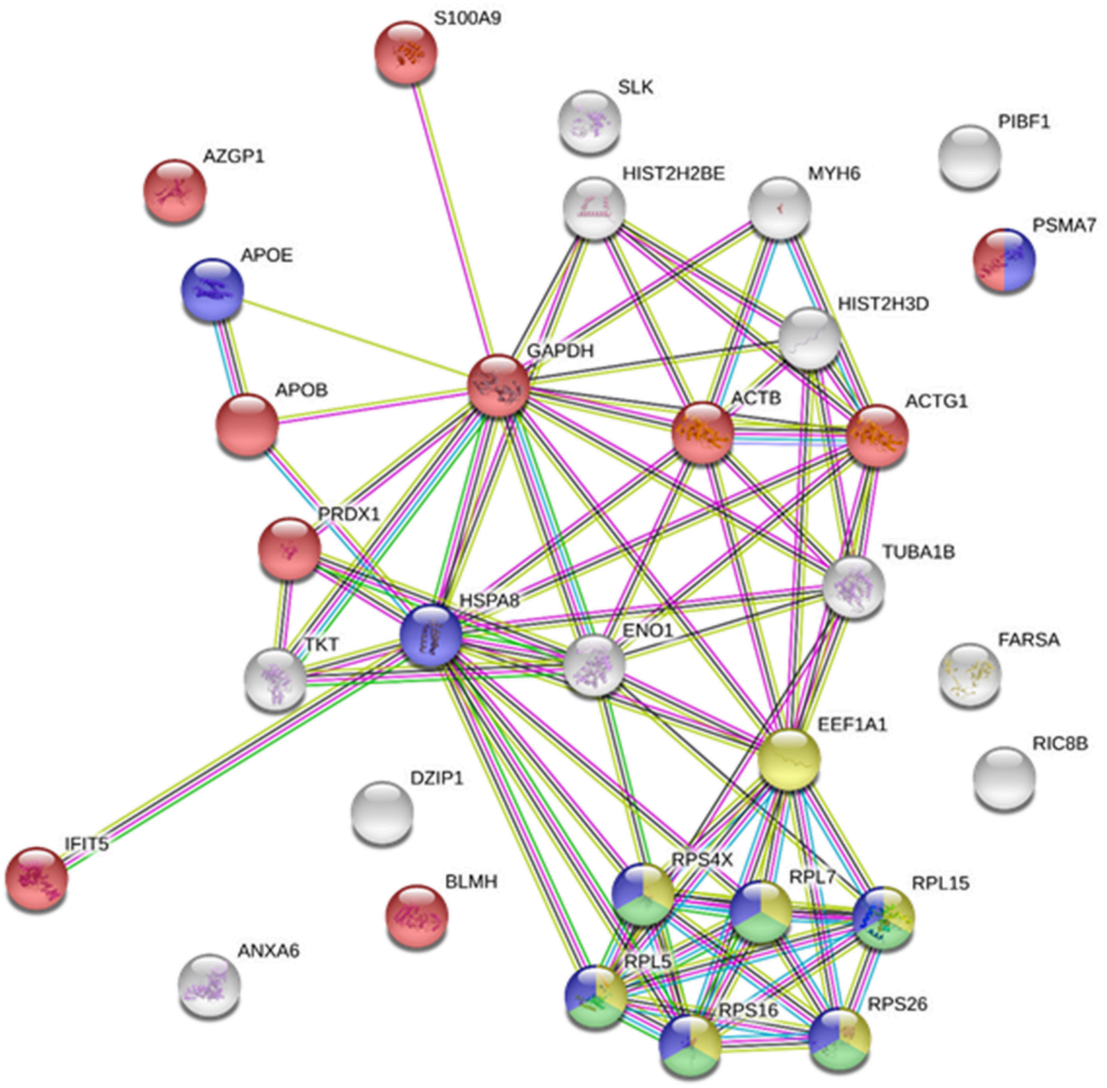
Immunoprecipitated proteins from RBCs exposed to VHSV. Lysates from RBCs exposed to VHSV at MOI 1 for 3 h were immunoprecipitated (IP) with monoclonal antibody against N protein of VHSV (2C9). The IP complex was precipitated with protein A Sepharose 4 Fast Flow beads. The obtained proteins were analyzed by mass spectrometry. A representation of constructed protein-protein interactions of IP proteins is shown. Nodes represent proteins, and edges denote the interactions between 2 proteins. Node colors indicate proteins functionally annotated with STRING software. Red, immune system process; blue, viral process; yellow, translation elongation; green, viral transcription.

It was recently reported that VHSV infection appeared to be halted in rainbow trout RBCs (4). However, specific RBC proteins that interact directly with VHSV have not been identified. To address this question, lysates of VHSV-exposed RBCs were immunoprecipitated with monoclonal antibody against N protein of VHSV because NVHSV is one of the major structural components of VHSV (43) and therefore is an ideal candidate to identify potential protein interactions. The resulting immune complexes were analyzed by mass spectrometry. A total of 31 proteins were identified in the immunoprecipitate (IP) of VHSV-exposed RBCs (Table 4; Supplementary Table 1). Importantly, the NVHSV protein was identified among the IP proteins (Supplementary Table 1). An interactome network was built for these proteins to reveal protein-protein interactions and predict functional associations using STRING software. As shown in Figure 1, 23 proteins highly interacted with each other, with a PPI enrichment *P* < 0.05, and 9 proteins did not show interaction. Predicted functional associations of these proteins were related to: (i) translation elongation (eukaryotic translation elongation factor 1 alpha [EEF1A1], ribosomal protein S4 X-linked [RPS4X], ribosomal protein L5 [RPL5], ribosomal protein L7 [RPL7], ribosomal protein L15 [RPL15], ribosomal protein S16 [RPS16], and ribosomal protein S26 [RPS26]); (ii) viral transcription (RPS4X, RPL5, RPL7, RPL15, RPS16, and RPS26); (iii) viral process (RPS4X, RPL5, RPL7, RPL15, RPS16, RPS26, apolipoprotein E [APOE], heat shock 70 kDa protein 8 [HSPA8], and proteasome [prosome, macropain] subunit alpha type 7 [PSMA7]); and (iv) immune system process (alpha-2-glycoprotein 1, zinc-binding [AZGPI], apolipoprotein B [including Ag(x) antigen, APOB], peroxiredoxin 1 [PRDX1], PSMA7, S100 calcium binding protein A9 [S100A9], glyceraldehyde-3-phosphate dehydrogenase [GAPDH], actin beta [ACTB], actin gamma 1 [ACTG1], bleomycin hydrolase [BLMH], and interferon-induced protein with tetratricopeptide repeats 5 [IFIT5]).

**Table 4:** Mass spectrometry proteomic analysis of immunoprecipitated proteins from VHSV-exposed RBCs.

**Main accession**	***Homo sapiens* ortholog** **gene symbol**	**Gene description**	**Mascot score**	**N° of peptide** **sequences identified**
A0A0B5E7Z4	ACTB	Actin beta	675	9
A0A146YS88	ACTG1	Actin gamma 1	63	1
W5N1H4	ANXA6	Annexin A6	53	1
P04114	APOB	Apolipoprotein B (including Ag(x) antigen	112	2
H0Y7L5	APOE	Apolipoprotein E	56	1
P25311	AZGP1	Alpha-2-glycoprotein 1, zinc-binding	87	1
J3KSD8	BLMH	Bleomycin hydrolase	55	1
H2TZ44	DZIP1	DAZ interacting protein 1	49	1
Q2TS51	EEF1A1	Eukaryotic translation elongation factor 1 alpha	170	3
C1J0I5	ENO1	Enolase 1	114	2
K7EPH2	FARSA	Phenylalanyl-tRNA synthetase, alpha subunit	54	1
C1BLG8	GAPDH	Glyceraldehyde-3-phosphate dehydrogenase	58	1
X1WG17	HIST2H2BE	Histone cluster 2, H2be	253	4
Q5GAW2	HIST2H3D	Histone cluster 2, H3d	58	1
A0A0C5BIF3	HSPA8	Heat shock 70kDa protein 8	140	2
A0A068FNU8	IFIT5	Interferon-induced protein with tetratricopeptide repeats 5	51	1
I6WP32	MYH6	Myosin, heavy chain 6	53	1
A0A1A7WCM1	PIBF1	Progesterone immunomodulatory binding factor 1	51	1
A0A060YDT1	PRDX1	Peroxiredoxin 1	64	1
A0A060XGK1	PSMA7	Proteasome (prosome, macropain) subunit, alpha type, 7	51	1
Q1JQ20	RIC8B	Resistance to inhibitors of cholinesterase 8 homolog B	53	1
A0A0S7EQ42	RPL15	Ribosomal protein L15	71	1
A0A060WXW6	RPL5	Ribosomal protein L5	59	1
A0A143WEA0	RPL7	Ribosomal protein L7	48	1
A0A0E9WET2	RPS16	Ribosomal protein S16	137	2
A0A0P7TLE4	RPS26	Ribosomal protein S26	57	1
A0A087XME4	RPS4X	Ribosomal protein S4, X-linked	51	1
P06702	S100A9	S100 calcium binding protein A9	75	1
A0A060W847	SLK	STE20-like kinase	51	1
M4A1M1	TKT	Transketolase	50	1
A0A060WX95	TUBA1B	Tubulin, alpha 1b	77	1

Among all identified proteins, we focused on IFIT5 because the IFIT family has recently emerged as an important player in antiviral innate immunity (12–18). To validate IFIT5 in the IP sample, a synthetic peptide was synthesized and a Nano LC ESI-MSMS analysis was performed. The synthetic peptide resulted in the same mass spectrum fragmentation obtained from IP peptide. Therefore, the synthetic peptide confirmed IFIT5 found by shotgun proteomics (Supplementary Figure 3).

### IFIT5 Gene and Protein Expression in Rainbow Trout RBCs After VHSV Exposure

**Figure 2:**
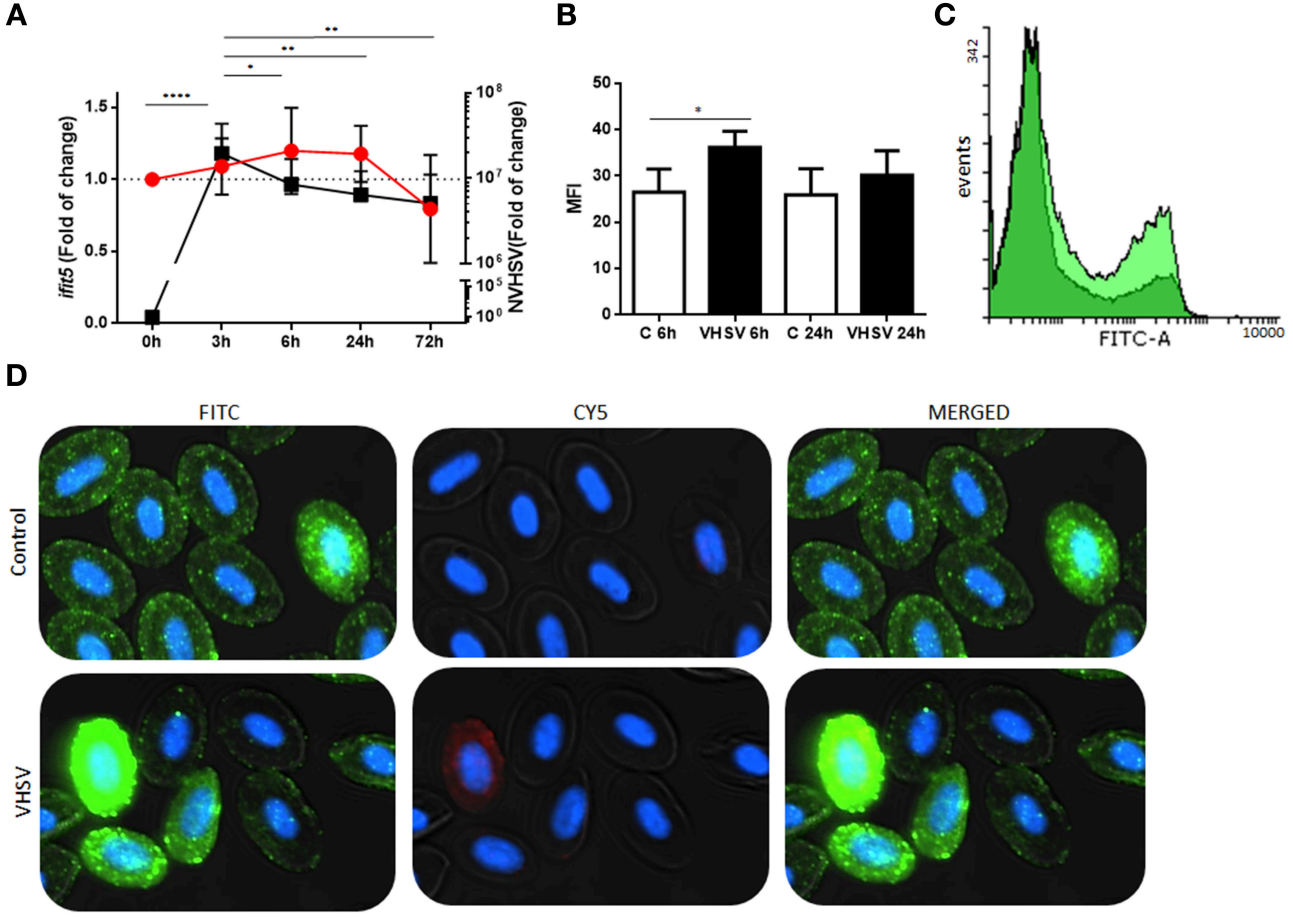
Time course of NVHSV gene replication and *ifit5* gene expression in VHSV-exposed rainbow trout RBCs. **(A)** Ficoll-purified RBCs were exposed to VHSV MOI 1 for different amounts of time (0, 3, 6, 24, and 72 hpe). The N gene of VHSV (NVHSV, black square) and *ifit5* (red circle) gene expression profiles were analyzed by RT-qPCR. Gene expression was normalized to the reference gene *ef1*α and relativized to control cells (RBCs unexposed to VHSV). Data represent the mean ± SD, *n* = 7. Tukey's multiple comparison test was performed for statistical analysis among all time points. ^*^, ^**^, and ^****^ indicate *P* < 0.05, < 0.01, and < 0.0001, respectively, for NVHSV. Lines indicate the trend followed by VHSV (black) and *ifit5* (red) during the time course assay. **(B)** IFIT5 protein expression observed in RBCs exposed to VHSV MOI 1 at 6 and 24 hpe were evaluated via flow cytometry using mouse anti-IFIT5 primary antibody and anti-mouse IgG CF^TM^ 488 as a secondary antibody. Mean fluorescent intensity (MFI) is shown. Data represent the mean ± SD, *n* = 4. Kruskal-Wallis Test with Dunn's multiple comparison *post-hoc* test was performed in comparison with control (^*^P < 0.05). **(C)** Representative flow cytometry overlay histograms showing unexposed RBCs (dark green) and VHSV-exposed RBCs at MOI 1 after 6 hpe (bright green). **(D)** Representative costaining immunofluorescence of IFIT5 and GVHSV in RBCs unexposed (control) or exposed to VHSV MOI 100, at 6 hpe, using antibodies against IFIT5 and GVHSV proteins. IFIT5 is stained green (anti-mouse IgG CF™ 488 secondary antibody), GVHSV is stained red (anti-rabbit IgG CF™ 647 secondary antibody), and nuclei are stained with DAPI. The image was taken at 60X magnification.

A time course assay was performed to determine gene and protein expression of rainbow trout IFIT5 in VHSV-exposed RBCs. Ficoll-purified RBCs were exposed to VHSV at MOI 1, and the expression levels of IFIT5 transcript and protein were evaluated at different time points by RT-qPCR and flow cytometry, respectively. VHSV replication in RBCs was evaluated at each time point by RT-qPCR using the N gene of VHSV. The *ifit5* gene transcripts started to increase by 3 hpe in RBCs, which correlated with the time point of the highest VHSV replication (Figure 2A). By 6 hpe, VHSV replication began to decrease, which coincided with the highest transcriptional and protein expression level of IFIT5, Figures 2A–D). After this time, IFIT5 transcripts and protein levels decreased (Figures 2A,B), and *ifit5* transcripts reached the basal level at 72 hpe (Figure 2A). Colocalization between IFIT5 and GVHSV was observed by immunofluorescence imaging (Figure 2D). A similar time course assay was performed in RTG-2 cells, another fish cell line (Supplementary Figure 4). *ifit5* gene expression in RTG-2 increased in VHSV-infected cells at 24 hpe, but this *ifit5* upregulation was not enough to decrease VHSV replication. At 72 hpe, the cell monolayer was destroyed.

### PLA Between IFIT5 and GVHSV

**Figure 3:**
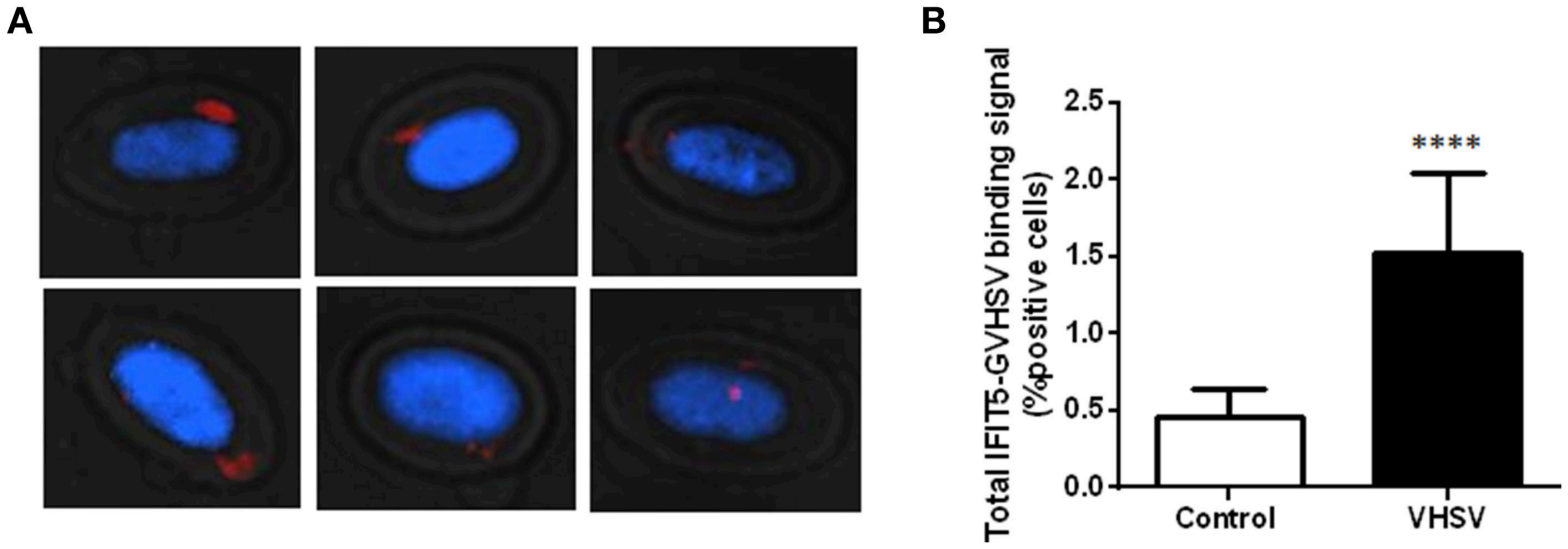
Imaging and quantification of PLA between IFIT5 and GVHSV. RBCs exposed to VHSV MOI 1 for 6 h were subjected to a PLA using specific antibodies against IFIT5 and protein G of VHSV. **(A)** Representative images of cells showing IFIT5-GVHSV colocalization (cells with red dots inside). Fluorescence images were taken at 60X magnification. Nuclei were stained with DAPI. **(B)** Graph representing the percentage of IFIT5-GVHSV-positive cells in the PLA. Data represent the mean ± SD, *n* = 3. A Mann-Whitney test was performed for statistical analysis. ^****^P < 0.0001.

To investigate possible interactions or proximity (proteins within 40 nm) between IFIT5 and VHSV, and to further corroborate the colocalization between IFIT5 and GVHSV observed by immunofluorescence (Figure 2D), a PLA was performed. We selected the GVHSV protein because it is the major surface protein of the virion. The highest IFIT5 expression level was observed at 6 hpe to VHSV, so we chose this time point to perform the PLA. Cells with red dots inside the cytoplasm were considered positive (Figure 3A). VHSV-exposed RBCs had significantly more positive cells than control RBCs (Figure 3B).

### Analysis of Compounds Docked to RNA-binding Pocket Cavity of IFIT5

**Figure 4:**
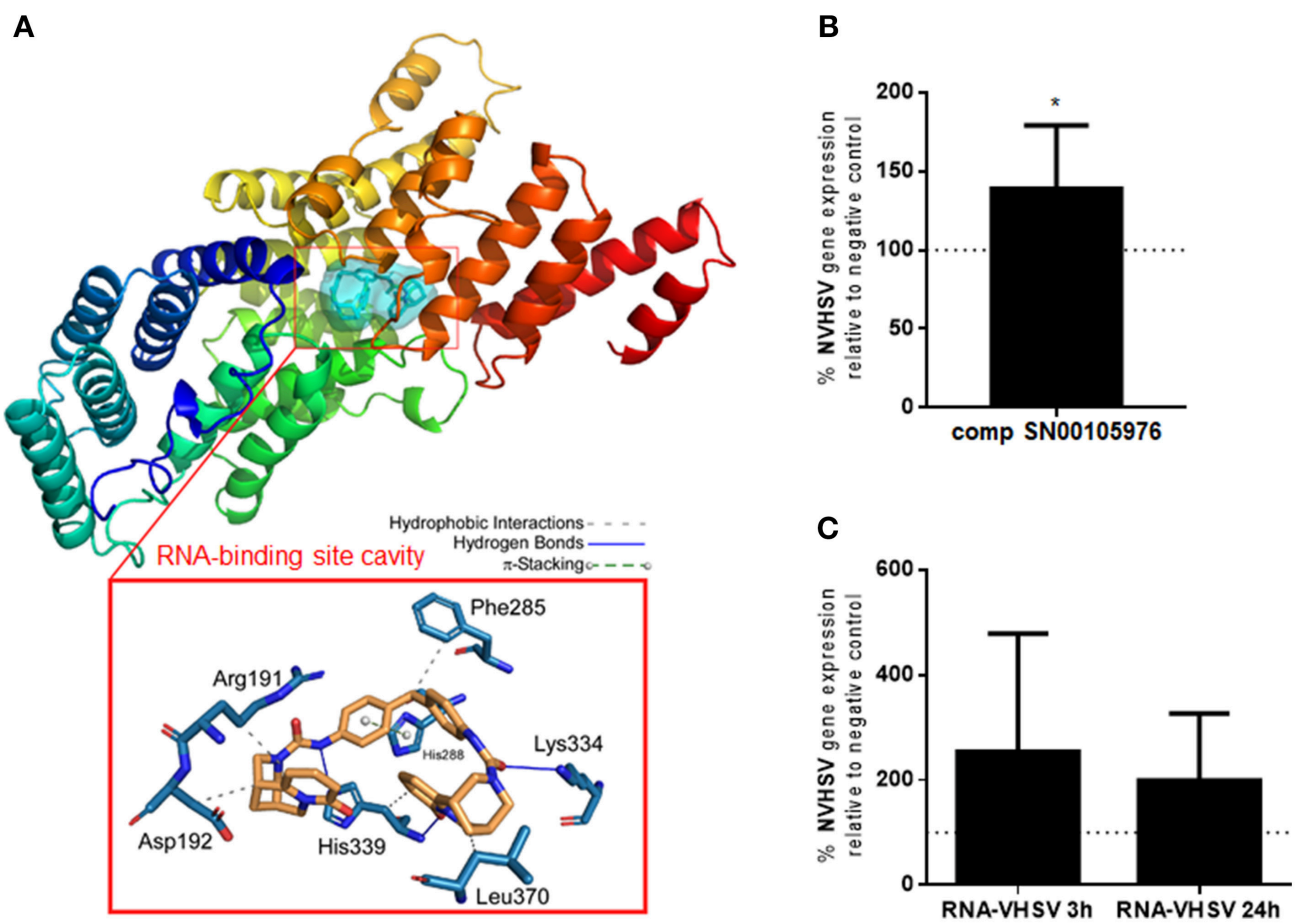
IFIT5 modulation increases VHSV replication. **(A)** Molecular docking analysis for the selected compound SN00105976 against IFIT5 RNA-binding pocket cavity. The secondary structure of the IFIT5 protein is shown from the N terminus (blue) to the C-terminus (red), and pictures the compound interacting in the IFIT5 RNA-binding pocket cavity. The compound and IFIT5 RNA-binding pocket-interacting residues of the binding site and each type of molecular interaction are expanded in the bottom red box. Interactions were detected with the Protein–Ligand Interaction Profiler (PLIP) algorithm (44). **(B)** RBCs incubated with 4,860 nM of compound SN00105976 for 24 h and exposed to VHSV MOI 1 for 24 h. The percentage of VHSV replication relative to VHSV-exposed RBCs (negative control) was determined by RT-qPCR of the NVHSV gene. Data represent the mean ± SD, *n* = 7. A Wilcoxon matched-pairs signed rank test was performed for statistical analysis. ^*^P < 0.05. **(C)** RBCs electroporated with VHSV RNA and incubated for 24 h before being exposed to VHSV MOI 1 for 3 or 24 h. Gene expression was normalized to the eukaryotic 18S rRNA gene. Percentage of VHSV replication relative to VHSV-exposed RBCs (negative control) was determined by RT-qPCR of the NVHSV gene. Data represent the mean ± SD, *n* = 3. A Wilcoxon matched-pairs signed rank test was performed for statistical analysis.

One of the established antiviral mechanisms of the IFIT family is recognition of viral mRNA (14–16) by the RNA-binding pocket cavity (28). Therefore, we searched for natural compounds that could bind to the IFIT5 RNA-binding pocket cavity and modulate its function. Natural compounds from the SuperNatural II database (19) were screened with a docking procedure. Starting from a library of 325,508 compounds, molecular docking experiments resulted in selection of compounds with a low Gibbs free energy variation value (ΔG, kcal/mol), and then of potentially higher affinity. We used the 3D model of rainbow trout IFIT5 (29) to carry out the molecular docking experiments at the RNA-binding pocket cavity. Up to 20 poses per compound bound to the explored RNA-binding site cavity (Figure 4A) were achieved. Overall, 0.16% (*n* = 507) of compounds had a ΔG ≤-11.5 kcal/mol. Additional filters were applied until a reasonable number of candidate compounds were obtained. We analyzed 13 ADMET parameters for each compound, and only compounds with values within the limits of each parameter as described by Ruiz-Torres et al. (31) passed through this screen. After ensuring commercial availability, 19 compounds were selected as candidates for IFIT5 modulators (Supplementary Table 2). The molecular structures of these compounds were compared, resulting in different clusters with up to 70% identity. This strategy allowed us to choose at least one representative compound of each cluster to experimentally test for IFIT5 modulating activity. Ultimately, 3 compounds (SN00105976, SN00110204, and SN00130855) were selected for experimental testing.

### Modulating IFIT5 Protein in RBCs

The percentage of viability in RBCs treated with the 3 selected potential IFIT5 modulator compounds (concentrations ranging from 540 nM to 4,860 nM) was >99.9% for compounds SN00105976 and SN00130855. We used these 2 compounds in additional assays to test their ability to modulate IFIT5 function. We evaluated the modulatory activity of the compounds on IFIT5 antiviral function in RBCs by exposing compound-treated RBCs to VHSV and evaluating VHSV replication. Only SN00105976 appeared to modulate IFIT5 antiviral function: RBCs incubated with this compound at 4,860 nM for 24 h showed a significant increase in VHSV replication (Figure 4B).

We also performed an indirect assay to modulate the IFIT5 RNA-binding pocket cavity by pretreating RBCs with VHSV RNA (presumably the IFIT5 target). We assumed that preincubating RBCs with VHSV RNA would result in competition with viral RNA for the IFIT5 binding pocket cavity and hence increase viral replication. As expected, RBCs electroporated with VHSV RNA showed an increase in VHSV replication (Figure 4C).

### Silencing *ifit5* Gene Expression

**Figure 5:**
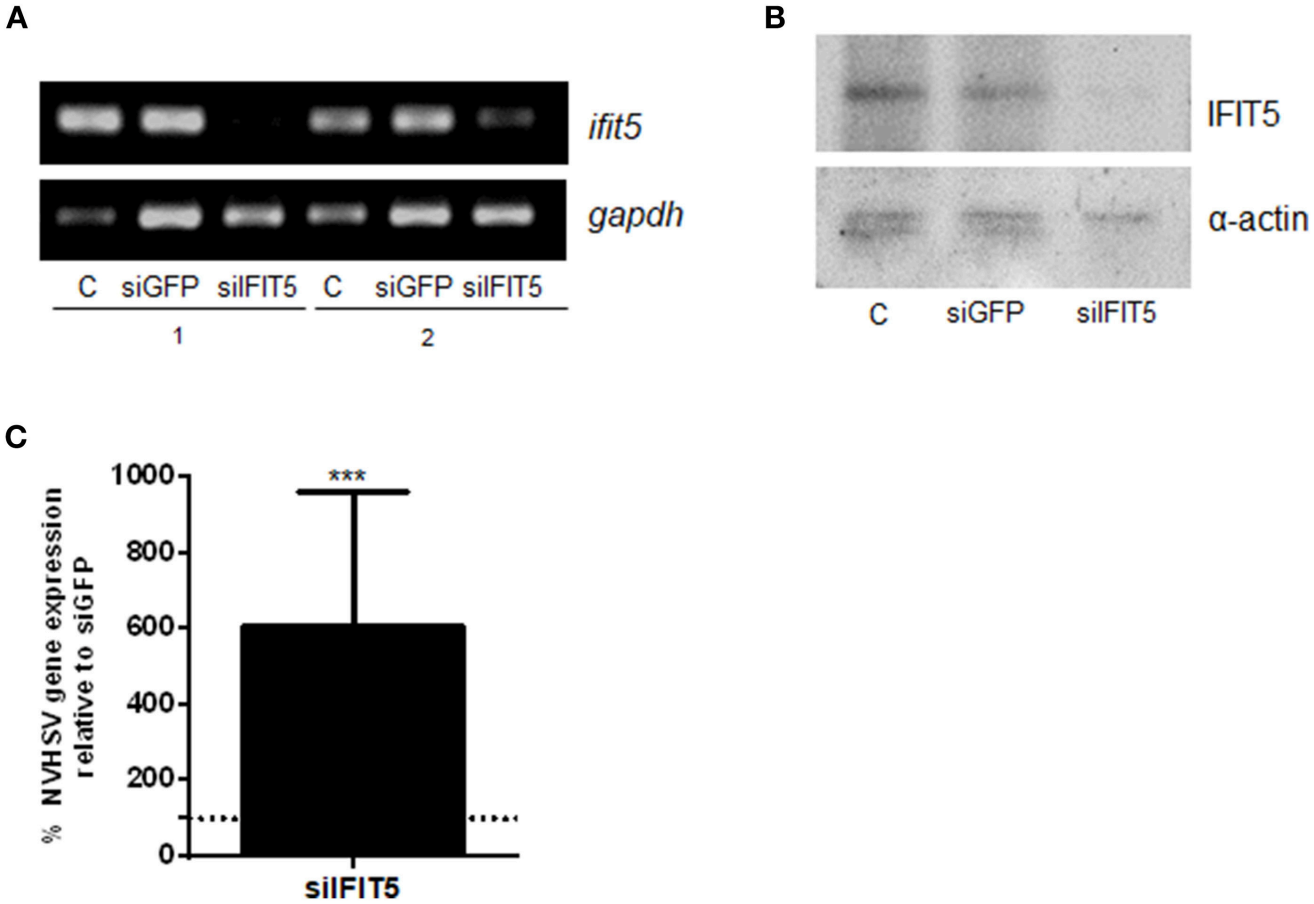
The effect of *ifit5* silencing on VHSV replication. RBCs electroporated with a mixture of 3 different *ifit5* siRNA sequences were incubated for 3 days. **(A)** *ifit5* gene silencing was evaluated by RT-PCR. siGFP was used as a negative control. The endogenous gene control was *gapdh*. C shows RBCs electroporated without RNA; 1 and 2 indicate the sample number. **(B)** Representative image of IFIT5 silencing at protein level by western blot using anti-IFIT5 antibody. The endogenous protein control was α-actin. **(C)** At 3 days after siRNA treatment, RBCs were exposed to VHSV MOI 1 for 24 h. The percentage of VHSV replication relative to the negative control was evaluated by RT-qPCR of the NVHSV gene. Gene expression was normalized to the reference eukaryotic 18S rRNA gene. Data represent the mean ± SD, *n* = 3. A Wilcoxon matched-pairs signed rank test was performed for statistical analysis. ^***^P < 0.001.

To further evaluate the role of IFIT5 in the antiviral mechanisms of rainbow trout RBCs, we performed *ifit5* gene silencing assays using siRNA. After silencing *ifit5*, its expression in RBCs decreased in RT-PCR experiments (Figure 5A) and in western blot assay (Figure 5B). We noted a significant increase in VHSV replication in RBCs pretreated with siIFIT5 sequences in RT-qPCR experiments (Figure 5C).

## Discussion

A wide variety of defense mechanisms have been reported for nucleated RBCs in response to VHSV exposure (4). To identify which rainbow trout RBC proteins interact directly with VHSV, we characterized the immunoprecipitated proteome of VHSV-exposed RBCs using an antibody against the N protein, which constitutes one of the major structural components of VHSV (43). The IP proteomic characterization included 31 proteins by mass spectrometry analysis. It should be taken into account that many of the proteins detected in the IP could have been immunoprecipitated due to a nucleation effect of protein-protein interactions in the protein milieu. However, an interactome network from the IP proteins revealed interesting functional associations, such as translation elongation, viral transcription, viral process, and immune system process. Similar cellular proteins have been found in dengue virus (DENV)-infected human hepatic cells (Huh-7) in a combined immunoprecipitation assay using anti-NS1 antibody from DENV and affinity chromatography assay (45). Among them, they identified GAPDH, EEF1A1, histones, heat shock proteins, PRDX, tubulin, and ribosomal proteins. Ribosomal proteins were the most abundant proteins identified (50%) (45). In our study, 20% of identified proteins were ribosomal proteins. The main function of the ribosomal proteins is mRNA translation. However, several ribosomal proteins have other functions, such as regulatory functions in fundamental processes related to the cell cycle, apoptosis, development, and oncogenes (46–48). It is well-known that viruses depend on ribosomes to synthesize proteins (49). Several ribosomal genes or proteins have a significant association with virus. For example, the ribosomal protein RPL18 interacts with the DENV NS1 protein and is required during its replicative cycle (45). RPL18 and RPL7 have been also found in siRNA screens performed with yellow fever and West Nile virus (50). In addition, RPL6, RPL3, and RPL15 were significantly associated with viral acute respiratory infection in infants (49). Moreover, RPS4X mRNA interacts with nonstructural protein 5B (NS5B) of hepatitis C virus (51). Also, vesicular stomatitis virus (VSV) mRNA translation depends specifically on the 60S ribosomal protein RPL40 (52). We also found glyceraldehyde 3-phosphate dehydrogenase, GAPDH, an important metabolic enzyme, which has been described to interact with some viruses such as Japanese encephalitis virus (53), human immunodeficiency virus type 1 (54, 55), cucumber mosaic virus (56), DENV (57), bamboo mosaic virus (58), hepatitis A virus (59), and human parainfluenza virus type 3 (60). Therefore, GAPDH is important in some viral infections, but we do not know how GAPDH interacts with VHSV nor the biological significance of this interaction.

Several proteins related to the immune system were identified in the IP proteomic characterization. We identified the S100A9 protein, which has been recently classified as a novel damage-associated molecular pattern, and has been associated with hepatitis B virus infection (61) and bovine viral diarrhea virus (BVDV), which interacts with BVDV Npro protein to decrease virus production (62). The involvement of S100A9 in rainbow trout RBC response to VHSV infection has not been studied and will be part of our future investigations. PRDX, or natural killer enhancing factor (NKEF), belongs to a family of antioxidant enzymes that has been described to protect against viral infection in fish (63). In VHSV-exposed RBCs, increased *nkef* transcripts were detected at 3 and 72 hpe (4). However, further studies are needed to determine the role of fish RBC NKEF in VHSV infection. This is part of our ongoing research.

Also related to the antiviral immune response, we identified the IFIT5 protein in the IP proteomic analysis. IFITs are a family of proteins with tetratricopeptide repeats induced after the production of type I interferon (8). These proteins have recently emerged as important players in antiviral innate immunity (12–18). IFIT genes have been identified for many species, including mammals and various birds, reptiles, amphibians, and bony fish (9). Until now, a wide repertoire of mechanisms have proposed as to how IFIT proteins restrict viral replication (64). Here, we showed a correlation between decreasing VHSV replication and the highest IFIT5 expression at 6 hpe in RBCs. However, in RTG-2 cells exposed to VHSV, there was no correlation with decreasing viral replication as occurs in RBCs despite upregulation of *ifit5* gene expression. This may be due to the high constitutive *ifit5* gene expression level that we found in RBCs compared with RTG-2 (Supplementary Figure 4). Some untreated RBCs showed intense cell staining with IFIT5 antibody in immunofluorescence experiments, which may also result from the high constitutive *ifit5* expression level in RBCs. Similar to IFIT5, another interferon-stimulated gene (ISG) protein such as Mx was reported to have a high basal expression level in RBCs (7). Therefore, high IFIT5 basal levels could be implicated in the early disappearance of VHSV. IFIT5 expression was not affected by the reported decrease of type I IFN production after VHSV exposure (4). Therefore, IFIT5 expression could be independent of IFN production, as has been described with Mx mRNA expression in rainbow trout injected with a plasmid encoding the chemokine CK5B (65). Proteomic sequencing of rainbow trout RBCs transfected with a plasmid encoding GVHSV revealed upregulation of antiviral mechanisms by ISG15. This was validated by the upregulation of *mx, pkr*, and *ifit5* gene expression, although *ifn1* gene expression appeared to be downregulated (40).

*ifit5* silencing resulted in a significant increase in VHSV replication in RBCs. Similar results were found with the generation of a chicken IFIT5 knockout fibroblast cell line by using the genome editing technology CRISPR/Cas9. Infection of this fibroblast IFIT5-ko cell line with RNA viruses such as Newcastle disease virus and VSV substantially supported viral replication in both cases (16). These results confirmed that IFIT5 is a crucial antiviral effector and that IFIT5 elimination weakens host barriers.

Mammalian IFIT5 also appears to have antiviral activity as an effector molecule by sequestration of viral RNA translation and through initiation of the innate immune response (66).

Upregulation of immune genes, such as *ifit5*, in Atlantic salmon after an intramuscular injection of IFNc results in protection against ISAV infection (67). Thus, it is possible that fish IFIT5, an ortholog of mammalian IFIT5 (8), participates in antiviral mechanisms. The crystal structure of human IFIT5 shows a RNA-binding pocket cavity, which supports the antiviral mechanism of sequestering viral RNA and dampening translation (28). This mechanism is used by IFIT proteins to bind viral mRNA and subsequently restrict viral replication (14–16). Here we show that modulating IFIT5 activity in rainbow trout RBCs, by using a chemical compound obtained by *in silico* molecular docking or with VHSV RNA, resulted in increased VHSV replication. These results indicated that rainbow trout IFIT5 may mediate antiviral activity in RBCs by binding viral RNA to the IFIT5 RNA-binding pocket cavity, thereby decreasing viral replication. This represents the first report of a fish IFIT5 antiviral mechanism. Similarly, chicken IFIT5 was recently described to specifically antagonize RNA viruses via this mechanism (16).

IFIT5 can also positively regulate innate immune responses in humans (13, 68, 69): ectopic expression of human IFIT5 upregulated the gene expression of IFN regulatory factor 3 (*irf3*) and nuclear factor-kB (*nf*κβ), whereas knockdown of IFIT5 impaired the transcription of these genes (68, 69). Nevertheless, non-significant differences were observed in the gene expression of Mx, IFNβ, viperin, interferon-induced 35 kDa (IFI35), and Arf-GAP with dual PH domain-containing protein 2 (ADAP2) proteins between transgenic and non-transgenic chicken that constitutively and stably expressed IFIT5 (66). However, previous studies of VHSV-exposed RBCs have shown downregulation of IFN1 protein and basal levels of Mx protein but upregulation of the cytokines interleukin 1 β (IL1β) and IL8, which are known to be activated by the transcription factor NFκβ (4). Therefore, an increase in these cytokines could be related to the NFκβ positive regulation by IFIT5. The role of rainbow trout IFIT5 in the activation of innate immune signaling pathways in RBCs needs to be further studied and is part of our ongoing research.

IFIT proteins can bind to viral proteins; for example, IFIT1 can bind to E1, a viral helicase of HPV, preventing viral replication in the nucleus (18). However, to our knowledge it has not been reported whether IFIT5 can interact directly with viral proteins. To this end, we investigated whether IFIT5 interacted with VHSV using a PLA. GVHSV protein was selected because it is the major virion surface protein and hence could increase the possibility of interaction with IFIT5. We found a significant increase in IFIT5-GVHSV colocalization-positive cells in VHSV-exposed RBCs compared to unexposed RBCs. More studies are needed to confirm an interaction between GVHSV and IFIT5, but it is clear that IFIT5 and VHSV are close enough to colocalize in the PLA.

In summary, we identified host proteins in rainbow trout RBCs that immunoprecipitated with VHSV. We focused on IFIT5 because it has recently emerged as an important player in antiviral innate immunity. We described a new defense mechanism based on IFIT5 antiviral activity in nucleated RBCs that appeared to contribute to halting the VHSV infection. This finding sheds light into novel antiviral therapeutics to mitigate the economic losses and social impact caused by viral infections in the aquaculture industry. This work broadens the knowledge of fish nucleated RBCs functions and their role in the immune response to viral infections.

## Ethics Statement

Experimental protocols and methods relating to experimental animals were reviewed and approved by the Animal Welfare Body and the Research Ethics Committee at the University Miguel Hernández (approval number 2014.205.E.OEP; 2016.221.E.OEP) and by the competent authority of the Regional Ministry of Presidency and Agriculture, Fisheries, Food and Water supply (approval number 2014/VSC/PEA/00205). All methods were carried out in accordance with the Spanish Royal Decree RD 53/2013 and EU Directive 2010/63/EU for the protection of animals used for research experimentation and other scientific purposes.

## Author Contributions

VC conceived ideas, performed experiments, analyzed data, and wrote the manuscript. MS-M and SP-M performed experiments. SC and MM performed proteomic sequencing. LM, IN, and FG produced antibodies. JE performed molecular docking analyses. MO-V conceived ideas, oversaw the research, analyzed data, and cowrote the manuscript. LP, JE, and JC contributed to the preparation of the manuscript.

### Conflict of Interest Statement

The authors declare that the research was conducted in the absence of any commercial or financial relationships that could be construed as a potential conflict of interest.
