# Supplementary material for: IFIT5 Participates in the Antiviral Mechanisms of Rainbow Trout Red Blood Cells
Source: Front Immunol. 2019 Apr 16;10:613. doi: 10.3389/fimmu.2019.00613 (PMC6476978; doi:10.3389/fimmu.2019.00613)
Supplement: Supplementary file 2 [file Table_2.DOCX]

**Supplementary Table S2**: Physicochemical and toxicological parameters calculated for the selected compounds against RNA-binding site of IFIT5 based on molecular docking analysis. Each cluster groups compounds with structures with up to 70% structural similarity. Compound names belong to the Super Natural II database (1). Compound in bold has been tested experimentally.

| Compound | ΔG, mean±S.D. (kcal/mol) | Cluster 70%^a^ | ^a^Total Surface Area, Å^2^ | ^a^TPSA, Å^2^ | cLogS^a^ | MW^a^ | cLogP^a^ | HBA^a^ | HBD^a^ | Ro5 violations^a^ | Druglikeness^a^ | DrugScore^a^ | RAT (LD50, mol/kg)^b^ | Caco-2 Permeability (LogPapp, cm/s)^b^ | TPT (pIGC50, µg/L)^b^ | FT (pLC50, mg/L)^b^ |
| --- | --- | --- | --- | --- | --- | --- | --- | --- | --- | --- | --- | --- | --- | --- | --- | --- |
| SN00100814 | -11.6 | 1 | 329.6 | 92.28 | -5.754 | 490.558 | 3.367 | 9 | 0 | 0 | 0.12440278 | -0.030797 | 2.6022 | 1.7413 | 0.5811 | 1.0971 |
| SN00037381 | -11.7 | 1 | 459.84 | 122.88 | -6.472 | 664.764 | 2.633 | 10 | 4 | 1 | 0.317839296 | 5.5108 | 2.5525 | 0.4506 | 0.4507 | 1.5785 |
| SN00105766 | -11.5 | 2 | 457.85 | 72.86 | -7.02 | 608.733 | 6.225 | 8 | 1 | 2 | 0.206887453 | 4.6261 | 2.6859 | 1.4829 | 0.6091 | 1.3321 |
| **SN00105976** | **-12** | **3** | **463.4** | **108.68** | **-3.1** | **630.75** | **7.362** | **10** | **2** | **2** | **0.1801296** | **4.3498** | **2.5277** | **0.4557** | **0.4845** | **1.5869** |
| SN00107934 | -11.7 | 1 | 393.98 | 84.94 | -8.805 | 554.6 | 4.459 | 7 | 1 | 1 | 0.165828597 | 3.7111 | 2.375 | 1.3479 | 0.4665 | 0.7562 |
| SN00108024 | -11.7 | 1 | 426.71 | 94.17 | -8.552 | 598.653 | 4.467 | 8 | 1 | 1 | 0.153390707 | 3.7748 | 2.5978 | 1.4101 | 0.5397 | 0.3081 |
| SN00108101 | -11.5 | 1 | 393.98 | 84.94 | -8.805 | 554.6 | 4.459 | 7 | 1 | 1 | 0.165828597 | 3.7111 | 2.375 | 1.3479 | 0.4665 | 0.7562 |
| SN00110065 | -11.6 | 1 | 421.73 | 111.24 | -8.761 | 598.609 | 4.101 | 9 | 1 | 1 | 0.0958061 | 3.53 | 2.359 | 1.2233 | 0.5361 | 0.6067 |
| SN00110174 | -11.8 | 1 | 364.32 | 91.65 | -6.01 | 539.01 | 2.422 | 7 | 2 | 1 | 0.206754385 | 0.52536 | 2.4581 | 0.9223 | 0.6757 | 0.9948 |
| SN00110204 | -11.9 | 1 | 364.32 | 91.65 | -6.01 | 539.01 | 2.422 | 7 | 2 | 1 | 0.2067544 | 0.52536 | 2.4581 | 0.9223 | 0.6757 | 0.9948 |
| SN00110480 | -11.5 | 1 | 355.5 | 78.51 | -7.061 | 556.458 | 3.615 | 6 | 2 | 1 | 0.198021669 | 3.5467 | 2.4249 | 1.0791 | 0.6052 | 1.3813 |
| SN00108482 | -11.6 | 4 | 427.5 | 65.18 | -8.902 | 576.906 | 6.801 | 4 | 2 | 2 | 0.089790322 | 1.3187 | 2.5269 | 1.1163 | 0.73 | 1.3466 |
| SN00109100 | -11.9 | 5 | 436.41 | 131.83 | -4.298 | 639.783 | 1.654 | 10 | 2 | 1 | 0.283544847 | 4.7456 | 3.3458 | 0.5455 | 0.5123 | 0.9771 |
| SN00109099 | -11.7 | 5 | 436.41 | 131.83 | -4.298 | 639.783 | 1.654 | 10 | 2 | 1 | 0.283544847 | 4.7456 | 3.3458 | 0.5455 | 0.5123 | 0.9771 |
| SN00111578 | -11.9 | 6 | 472.26 | 23.55 | -7.209 | 612.983 | 6.902 | 3 | 0 | 2 | 0.100618753 | 4.3303 | 2.8246 | 1.2447 | 0.7136 | 0.9799 |
| SN00113052 | -11.6 | 7 | 406.58 | 106.74 | -7.317 | 722.021 | 6.773 | 7 | 0 | 2 | 0.056084823 | -10.035 | 2.6194 | 0.8692 | 0.6035 | 1.3033 |
| SN00115843 | -12.2 | 8 | 454.76 | 84.96 | -4.364 | 621.719 | 7.319 | 9 | 0 | 2 | 0.267592035 | 7.42 | 2.5746 | 0.7198 | 0.5291 | 0.931 |
| SN00116397 | -11.7 | 3 | 483.3 | 140 | -1.646 | 792.883 | 6.424 | 13 | 0 | 3 | 0.082092982 | -7.3332 | 2.5055 | 0.7615 | 0.4771 | 1.0994 |
| SN00130855 | -11.7 | 9 | 278.18 | 59.92 | -9.14 | 408.42 | 6.528 | 4 | 0 | 1 | 0.0560286 | -2.0436 | 2.1916 | 1.1746 | 0.6702 | 2.0373 |

**Abbreviations:** Topological polar surface area (TPSA); molecular weight (MW); the calculated logarithm (base 10) of the solubility measured in mol/liter (cLogS); calculated logarithm of partition coefficient between n-octanol and water (cLogP); number of hydrogen bond donors (HBD); number of hydrogen bond acceptors (HBA); violation of Lipinski’s rules (Ro5 violations), FT, Fish Toxicity; TPT, *Tetrahymena Pyriformis* Toxicity; RAT, Rat Acute Toxicity. LD50 is the amount of a compound, given all at once, which causes the death of 50% (one half) of a group of test rats.

^a^ These parameters were calculated using the DATAWARRIOR software v4.7.2 (2).

^b^ These parameters were calculated using the <http://lmmd.ecust.edu.cn:8000/predict/> site [4].

**References**

1. Banerjee, P.; Erehman, J.; Gohlke, B.O.; Wilhelm, T.; Preissner, R.; Dunkel, M. Super Natural II--a database of natural products. Nucleic Acids Res 2015, 43, D935-939.

2. Sander, T.; Freyss, J.; von Korff, M.; Rufener, C. Datawarrior: An open-source program for chemistry aware data visualization and analysis. J Chem Inf Model 2015, 55, 460-473.

3. Cheng, F.; Li, W.; Zhou, Y.; Shen, J.; Wu, Z.; Liu, G.; Lee, P.W.; Tang, Y. AdmetSAR: A comprehensive source and free tool for assessment of chemical ADMET properties. J Chem Inf Model 2012, 52, 3099-3105.
