# Supplementary figures and images for: IFIT5 Participates in the Antiviral Mechanisms of Rainbow Trout Red Blood Cells
Source: Front Immunol. 2019 Apr 16;10:613. doi: 10.3389/fimmu.2019.00613 (PMC6476978; doi:10.3389/fimmu.2019.00613)

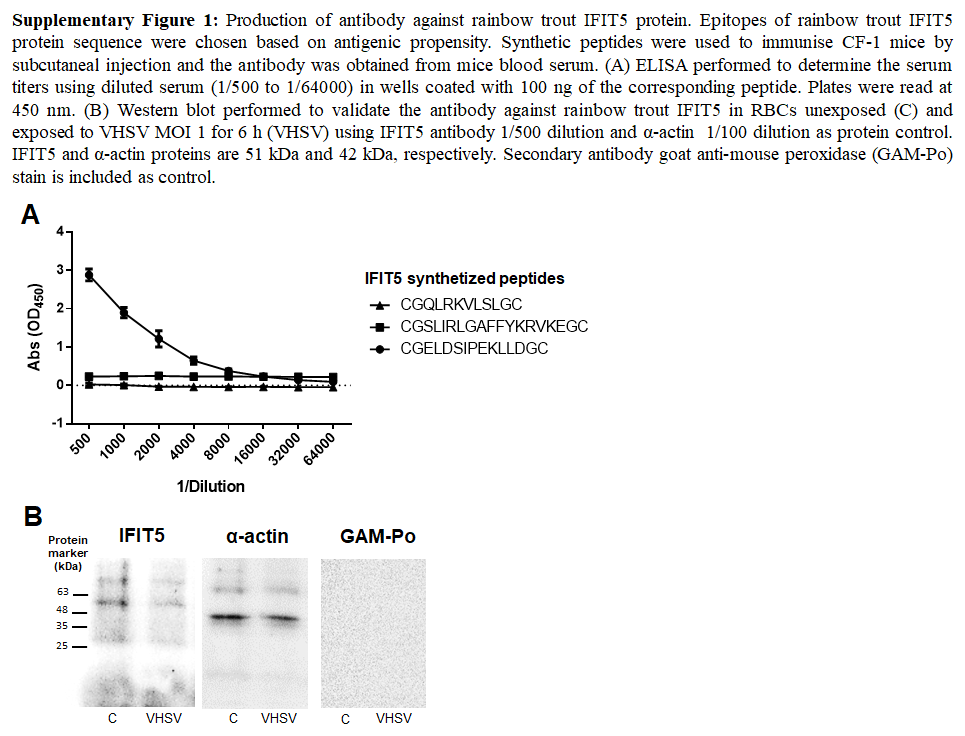

Supplement: Supplementary file 3 [file Image_1.TIF]

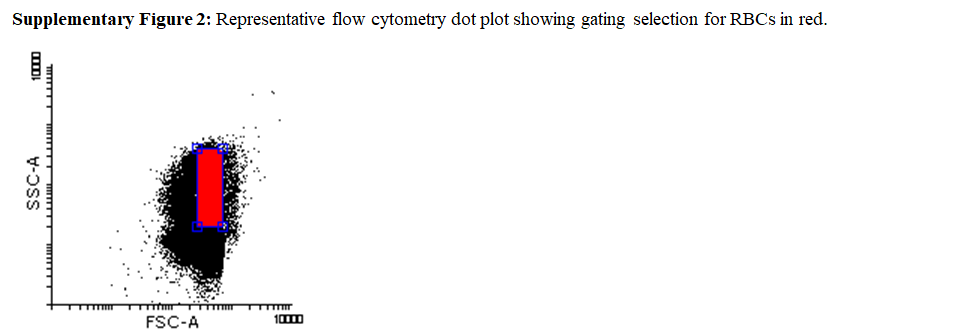

Supplement: Supplementary file 4 [file Image_2.TIF]

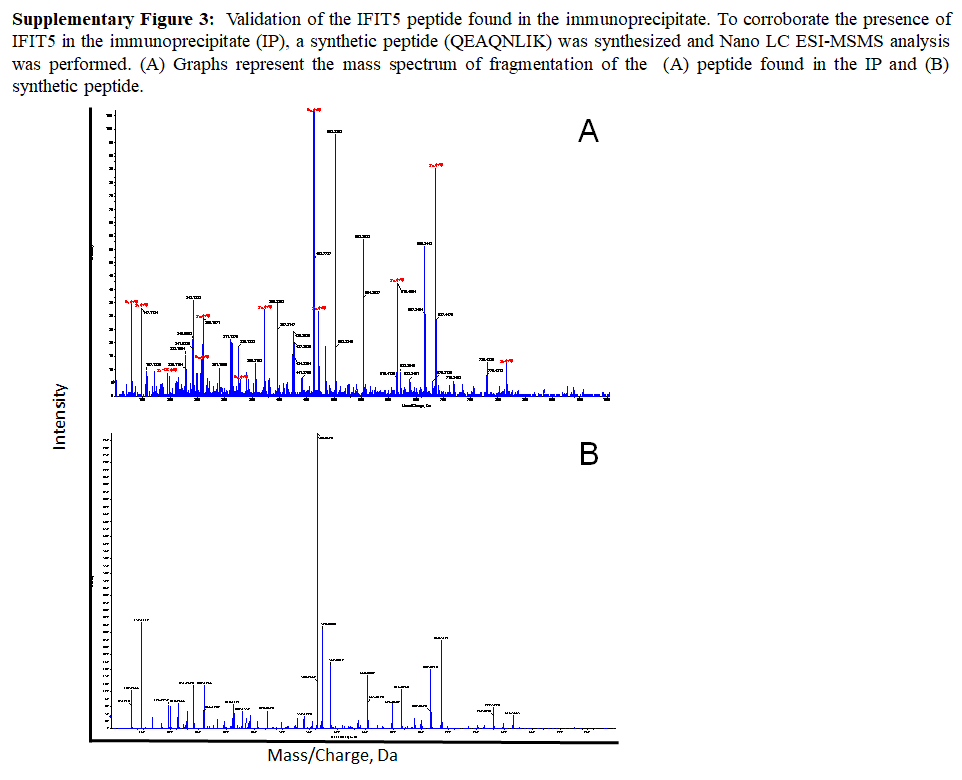

Supplement: Supplementary file 5 [file Image_3.TIF]

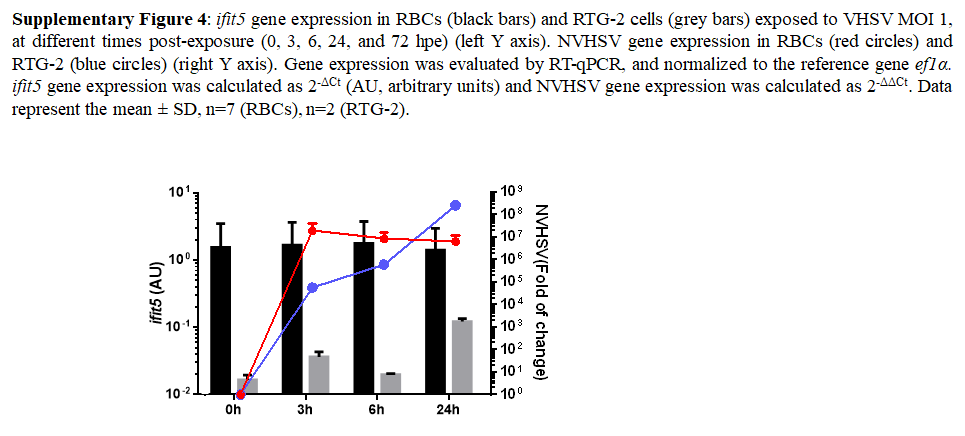

Supplement: Supplementary file 6 [file Image_4.tif]
